# Physiological, Biochemical and Transcriptomic Analysis of the Aerial Parts (Leaf-Blade and Petiole) of *Asarum sieboldii* Responding to Drought Stress

**DOI:** 10.3390/ijms222413402

**Published:** 2021-12-13

**Authors:** Fawang Liu, Tahir Ali, Zhong Liu

**Affiliations:** School of Pharmacy, Shanghai Jiao Tong University, Shanghai 200240, China; fawang90@126.com (F.L.); Tahirali@sjtu.edu.cn (T.A.)

**Keywords:** drought, *Asarum sieboldii* Miq., transcriptome, methyleugenol, safrole

## Abstract

*Asarum sieboldii* Miq. is a leading economic crop and a traditional medicinal herb in China. Leaf-blade and petiole are the only aerial tissues of *A. sieboldii* during the vegetative growth, playing a vital role in the accumulation and transportation of biomass energy. They also act as critical indicators of drought in agricultural management, especially for crops having underground stems. During drought, variations in the morphology and gene expression of the leaves and petioles are used to control agricultural irrigation and production. Besides, such stress can also alter the differential gene expression in these tissues. However, little is known about the drought-tolerant character of the aerial parts of *A. sieboldii*. In this study, we examined the physiological, biochemical and transcriptomic responses to the drought stress in the leaf blades and petioles of *A. sieboldii*. The molecular mechanism, involving in drought stress response, was elucidated by constructing the cDNA libraries and performing transcriptomic sequencing. Under drought stress, a total of 2912 and 2887 unigenes were differentially expressed in the leaf blade and petiole, respectively. The detection of many transcription factors and functional genes demonstrated that multiple regulatory pathways were involved in drought tolerance. In response to drought, the leaf blade and petiole displayed a general physiological character, a higher SOD and POD activity, a higher MDA content and lower chlorophyll content. Three unigenes encoding POD were up-regulated, which can improve POD activity. Essential oil in petiole was extracted. The relative contents of methyleugenol and safrole in essential oil were increased from 0.01% to 0.05%, and 3.89% to 16.97%, respectively, while myristicin slightly reduced from 24.87% to 21.52%. Additionally, an IGS unigene, involved in eugenol biobiosynthesis, was found up-regulated under drought stress, which was predicated to be responsible for the accumulation of methyleugenol and safrole. Simple sequence repeats (SSRs) were characterized in of *A. sieboldii*, and a total of 5466 SSRs were identified. Among them, mono-nucleotides were the most abundant repeat units, accounting for 44.09% followed by tri-, tetra-, penta and hexa-nucleotide repeats. Overall, the present work provides a valuable resource for the population genetics studies of *A. sieboldii*. Besides, it provides much genomic information for the functional dissection of the drought-resistance in *A. sieboldii*, which will be useful to understand the bio-regulatory mechanisms linked with drought-tolerance to enhance its yield.

## 1. Introduction

*Asarum sieboldii* Miq. is a perennial herb of the family Aristolochiaceae. It is mainly distributed in central and eastern China continent, covering south Shaanxi, north Sichuan and Chongqing, west and east Hubei, northwest Hunan, southwest Henan, west and southeast Anhui, northwest Jiangxi, northwest Zhejiang, and east Shandong, as well as in Japan and Korea [1,2,3,4]. In Chinese clinical practice, *A. sieboldii* is used as an antitussive and analgesic agent to treat cold, fever, aphthous stomatitis, toothache, gingivitis, and rheumatoid arthritis, wherein it has become an integral part of single or multi-herbal formulations for nearly two thousand years [5]. The main phytochemical and bioactive component of *A. sieboldii* is essential oil, which consists of diverse volatile compounds such as methyleugenol, asarylketone, cineol, safrole, limonene, eucarvone, and pellitorin [6]. Methyleugenol is one of the most important constituents of *A. sieboldii*, playing a variety of pharmacological roles in analgesic, antitussive, anti-asthmatic, anaphylactic, antihypertensive and insecticidal activities, whereas, safrole and myristicin are supposed to be genotoxic and carcinogenic substances [7,8,9]. Besides, methyleugenol is also found in various aromatic plants that are widely used in food products as flavoring agent [10,11]. Because of the increasing demand of crude drug market, *A. sieboldii* has been cultivated as a primary economic crop in Ningqiang county, southwest Shaanxi in China [12].

Drought, one of the most important abiotic stresses, plays an adverse role in plant growth. It has been demonstrated that drought can influence a series of physiological and molecular processes, and cause considerable losses in agricultural crop production [13,14,15]. Consequently, the response and adaptation of plants to drought stress has become a focal point of the research in recent decades [16]. Researchers have studied the regulation ability of several plants in response to drought stress, and have found a range of physiological and biochemical changes that enable plants to respond or adapt to the extreme environment [17]. For example, essential oils can be synthesized and stored in surface glandular trichomes in leaves, stems, and flowers in several plants [18,19]. However, environmental stress such as drought can significantly affect the content of essential oils and produce higher levels of specialized metabolites by regulating the multiple genes [20,21]. It has been known that appropriate abiotic stresses can increase the amount of the essential oils in *Ocimum basilicum*, *O. basilicum* and *O. americanum* [22]. Conversely, severe water deficiency can evidently damage the structure of plant cells and tissues, causing subsequent changes in phenotype and alteration in the physiological functions and productivity. For example, in Geranium (*Pelargonium hortorum*), severe drought caused a reduction in the number of flowers per plant [23]. In *Allocasuarina luehmannii*, the seedlings growth was normal under moderate drought. However, under severe drought, ascorbate and glutathione concentrations remained unaffected by drought treatments, but ascorbate became more oxidized under severe stress. Additionally, severe drought stress (SS) decreased stomatal conductance and net CO2 assimilation rates to ∼5% and ∼15% of the control values, respectively, and caused increases in internal CO2 concentration and photosystem II excitation pressure (1−qP), as well as decreases in water potentials, effective quantum yield of PSII (ΦPSII), maximum efficiency of PSII centers (Fv/Fm) and Fv′/Fm [24]. In *Arabidopsis thaliana*, severe drought affected reproductive development via inhibiting blooming, decreasing pollination, and causing abnormal development in anther and filament [25]. 

Because the essential oils were mainly deposited in the underground parts, i.e., root and rhizome, which were the harvest and useful part of cultivated *A. sieboldii* plants, we focused our attention to investigating the response of root and rhizome of *A. sieboldii* to drought stress in our previous research [26]. We found that the total essential oil content was reduced, however, methyleugenol content was improved, and several genes related to methyleugenol biobiosynthesis such as *HCT*, *CCR* and *IGS* were up-regulated. On the contrary, leaf blade and petiole are the vegetative aerial part of *A. sieboldii* individual, having vital roles in biomass and energy production. In agriculture, especially for those crops whose stems are under ground, the response of the aerial part of the crop to drought could be helpful to control the agricultural irrigation to reduce product loss. For example, potato is a drought-sensitive crop; due to its drought vulnerability has been mainly attributed to its shallow root system and low capacity of recuperation following a period of water stress. The physiological index such as chlorophyll content can be measured using multispectral imaging (bands 550, 710 and 810 nm) to measurement of drought tolerance, and the detected results can guide irrigation [27]. Unfortunately, so far, the effect of drought stress on the essential oils in leaf blade and petiole of *A. sieboldii* has not been reported. Additionally, the expression pattern of the genes involved in the essential oil biobiosynthesis in leaf blade and petiole of *A. sieboldii* under drought was also rarely known. 

Studying differentially expressed drought-resistant genes can provide the foundation for a deeper understanding of the molecular mechanisms underlying adaptation to drought, and many drought-resistance genes have been introduced into various crops, such as *OsDREB1A/1B/1F* and *OsWRKY11* in rice, *ZmPLC1* in maize, *soyBiP D* in soybean, and *hpAtPARP1* in canola [28,29,30,31,32,33]. The lack of genomic resources of *A.* *sieboldii*, including a reference genome, has impeded the development of unigenes to facilitate genetic studies. High-throughput RNA sequencing (RNA-seq) is an effective large-scale method to identify changes in gene expression patterns in response to stress conditions and is widely used in many plants, such as poplar, rice, and tomato [34,35]. Therefore, RNA-seq represents an optimal strategy to generate sequences that can be used with a robust de novo assembly method. 

In this study, to reveal the morphological, physiological and genetic variations associated with drought, we carried out an extensive sequencing analysis of the drought-stressed *A. sieboldii*, using the Illumina Hiseq 2000 platform. The basic plant physiology index responded to drought was detected, including the chlorophyll content, the MDA (malondialdehyde) content and the activities of antioxidant enzymes, i.e., SOD (superoxide dismutase) and POD (peroxidase). The quantity of methyleugenol, safrole and myristicin in essential oil was detected; the DEGs (differentially expressed genes) in methyleugenol biobiosynthesis pathway were analyzed. Furthermore, DEGs encoding transcription factors and DEGs in photobiosynthesis, chlorophyll content and plant hormone signal transduction pathway were analyzed. The SSR (simple sequence repeat) markers were searched, which provided useful genetic diversity information for *A. sieboldii*. Therefore, the evidence of this study has supplied sufficient genomic resources, which can contribute significantly to the discovery and annotation of unigenes, identify drought-related genes, and illuminate the relationship between drought stress and key genes involved in methyleugenol biobiosynthesis in *A. sieboldii*.

## 2. Results

### 2.1. Physiological Evaluation of A. sieboldii in Response to Drought 

**Figure 1:**
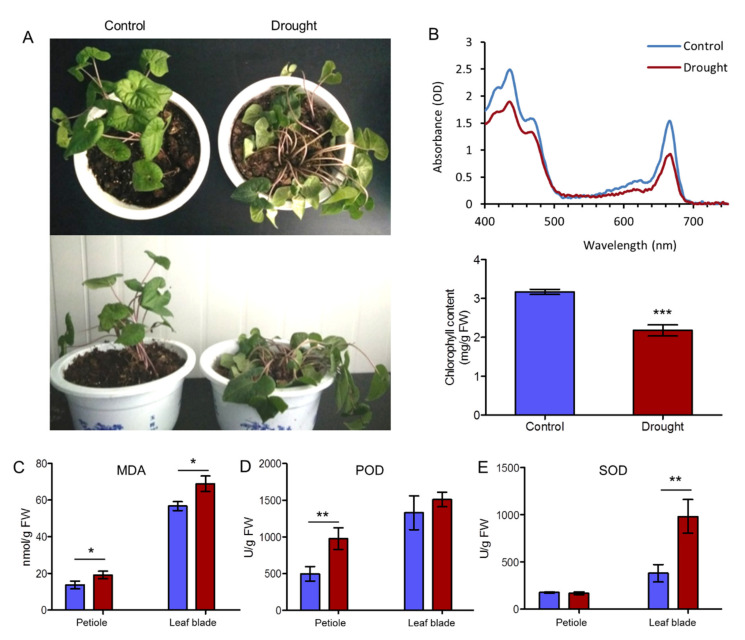
Effects of drought stress on phenotype, POD and SOD activity, chlorophyll and MDA contents of *A. sieboldii*. (**A**) The phenotypic change of *A. sieboldii* under drought treatment. (**B**) Chlorophyll content detected using UV absorption spectrometry method. (**C**) The MDA content. (**D**) The activities of POD. (**E**) The activities of SOD. Statistical analyses were conducted with Student’s *t*-test. Values were the mean ± SD of three replicates. Asterisks indicated significant differences at *p* < 0.05 (*), *p* < 0.01 (**), and *p* < 0.001 (***), respectively.

In drought treatment, the leaf blades withered and became smaller, and the petioles fell down (Figure 1A). The chlorophyll biosynthesis was significantly affected, and the decomposition of chlorophyll was speeded up, thus causing a decrease in chlorophyll content in the leaves. Using spectroscopic analysis, the contents of chlorophyll a and b were detected. The total chlorophyll content in the fresh leaf blades of normal growing *A. sieboldii* was 3.169 ± 0.067 mg/g fresh weight (F.W) but decreased to 2.178 ± 0.145 mg/g F. W after drought (Figure 1B). While MDA contents in leaf blade and petiole were both increased, a higher content was observed in leaf blade than in petiole, as MDA was the product of the lipid membrane peroxidation. This result indicated that drought had a heavier damage in leaf blade (Figure 1C). The enzyme activities of POD and SOD also increased in drought, of which the SOD activity varied from 380.79 U/g FW to 982.17 U/g FW while the POD activities improved from 1328.83 U/g FW to 1509.97 U/g FW in leaf blade and from 497.71 U/g FW to 977.72 U/g FW in petiole, promoting to eliminate active oxygen radicals, to protect the lipids from peroxidation, to delay plant senescence, and to maintain normal growth and development. The increase in POD and SOD activities indicated a self-protective mechanism of *A. sieboldii* against drought damage (Figure 1D,E). 

### 2.2. Essential Oil Accumulation in A. sieboldii under Drought Conditions

**Figure 2:**
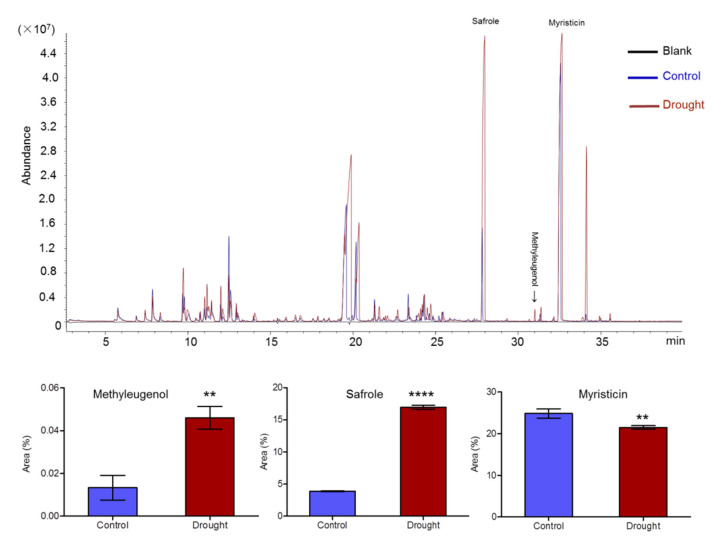
GC-MS analysis of methyleugenol, safrole and myristicin contents of *A. sieboldii* in drought stress. Statistical analyses were conducted with Student’s *t*-test. Values represented the mean ±SD of three replicates. Asterisks indicated significant differences at *p* < 0.01 (**) and *p* < 0.0001 (****), respectively.

Essential oil of *A. sieboldii* has been known mainly consisted of volatile phenylpropanoid compounds including methyleugenol, safrole, and myristicin, which are produced in phenylpropanoid biosynthesis pathway. The contents of methyleugenol (rt 30.671 min), safrole (rt 27.828 min) and myristicin (rt 32.574 min) in petioles were determined by comparing mass data of each peak with the National Institute of Standards and Technologies (NIST) database [36], and the matching values were more than 96% for each compound. The relative contents of methyleugenol, safrole and myristicin were 0.01%, 3.89% and 24.87%, respectively, in the control group. However, in the drought treated group, the contents of methyleugenol and safrole were significantly increased to 0.05% and 16.97%, while that of myristicin was slightly reduced to 21.52% (Figure 2). These results indicated that drought-stress induced the accumulation of medicinal compound methyleugenol, also the toxic compound safrole, but inhibited the biosynthesis of myristicin.

### 2.3. Comparative Transcriptome Analysis of A. sieboldii under Normal and Drought Treatments

#### 2.3.1. Transcriptome Sequencing and de Novo Assembly 

A total of 25.85 Gb clean data were obtained after removing reads that were of low-quality or contained adapter. For each sample, the clean data reached 6.24 Gb, and the Q30 was above 94.05%. The clean data were de novo assembled into contigs, which were further merged to generate 150,675 transcripts and 45,016 unigenes. The clean data were compared with the assembled transcript or unigene libraries. The mapped ratio was more than 84.21%. The total lengths of transcripts and unigenes were 218,185,217 bp and 44,921,544 bp, respectively. Mean sizes of total transcripts with N50 were in length of 2097 bp, while mean sizes of unigenes with N50 were in 1545 bp. The mean lengths of total transcripts and unigenes were 1448.05 bp and 997.90 bp, respectively. All unigenes were longer than 300 bp; 18,524 (41.15%) unigenes were between 300 and 500 bp; 12,228 (27.16%) were between 500 and 1000 bp; 8635 (19.18%) were between 1 and 2 kb; 5629 (12.50%) unigenes were over 2 kb (Table 1).

#### 2.3.2. Function Annotation 

**Figure 3:**
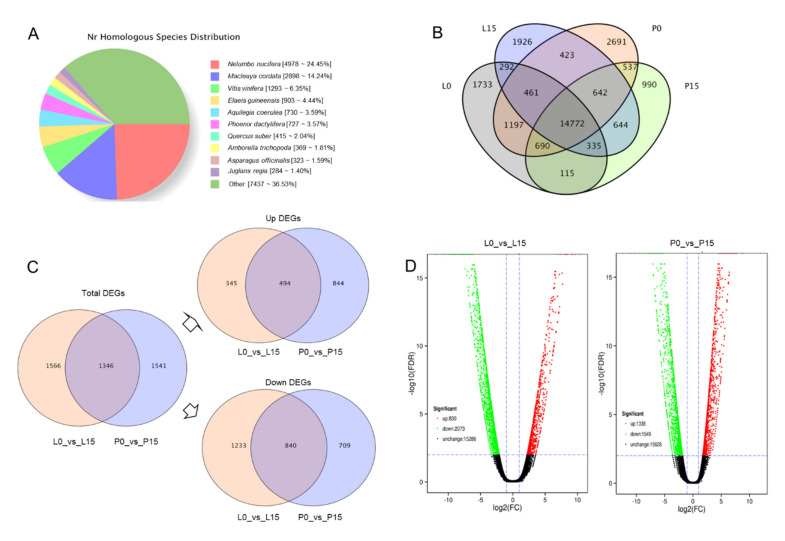
NR distribution in species, Venn diagram and Volcano plot of unigenes. (**A**) NR distribution of all unigenes in species. (**B**) Venn diagram of all unigenes. (**C**) Venn diagram of DEGs. (**D**) Volcano plot of DEGs. L0 and L15, leaf blades in control and drought for 15d; P0 and P15, petioles in control and drought for 15d.

A total of 20,551 unigenes had significant hits in eight public databases with an E-value < 10^−5^ for BLAST and E-value < 10^−10^ for HMMER. Among these unigenes, 20,366 (99.09%) unigenes were annotated with the nr database, followed by eggNOG (19,257, 93.70%), Swissprot (14,415, 70.14%), Pfam (14,251, 69.34%), KOG (12,185, 59.29%), GO (11,184, 54.42%), KEGG (7574, 36.85%), and COG (6226, 30.29%) (Table 2). In species distribution, 13,114 unigenes (63.47%) shared high similarity with ten plant species in nr database; however, the other 7437 unigenes (36.53%) could not find any hits in the database. Among these attributed unigenes, 4978 unigenes (24.45%) had variable numbers of hits with *Nelumbo nucifera*, followed by *Macleaya cordata* (2898, 14.24%), *Vitis vinifera* (1293, 6.35%), etc. (Figure 3A). Venn diagram of unigenes in different tissues was conducted, showing that while 14,772 unigenes were shared in all the four tissue samples, 15,860 unigenes were shared in L0 (leaf blade in control) and L15 (leaf blade in drought), and 16,641 unigenes were shared in P0 (petiole in control) and P15 (petiole in drought) (Figure 3B). 

#### 2.3.3. Analysis of DEGs

The parameters log_2_FC ≥ 2 and FDR ≤ 0.01 were set and used as the screening condition of DEGs. A total of 4453 DEGs were identified in L0_vs_L15 and P0_vs_P15 (Table S1). Among them, 1346 DEGs were found shared in both groups, including 494 up-regulated unigenes and 840 down-regulated unigenes (Figure 3C). 

Among the up-regulated DEGs, c26922.graph_c0, which was an uncharacterized protein, showed the highest fold change with a value of 8.83 (log2FC) in petiole, whereas c59969.graph_c0 that was annotated as paragine synthetase showed a most significant fold change with a value of 10.47 in leaf blade; and c45955.graph_c0, which contained NAC domain and encoded dehydration-responsive element-binding protein, showed a higher value both in leaf blade (log2FC value of 9.20) and petiole (log2FC value of 8.63). The DEGs in leaf blade and petiole with a prominent fold change were listed in Table S2. Among the 840 down-regulated unigenes, in petiole, c50544.graph_c0 showed a highest log2FC value of −12.34, followed by hydrophobic seed protein c22620.graph_c0. In leaf blade, c50544.graph_c0 also showed the highest fold change, followed by c22564.graph_c0 (Carbonic anhydrase), c57668.graph_c4 (UDP-glycosyltransferase), and c24185.graph_c0 (galactinol synthase). Other DEGs with higher fold change were listed in Table S3. 

We also made comparison among DEGs in different tissues. 2887 DEGs were found in petiole (P0_vs_P15), including 1338 up-regulated unigenes and 1549 down-regulated unigenes. 2912 DEGs were found in leaf blade (L0_vs_L15), including 839 up-regulated unigenes and 2073 down-regulated unigenes. It was clearly revealed that drought stress down-regulated more genes in leaf blade, but up-regulated more genes in petiole (Figure 3D).

#### 2.3.4. GO and KEGG Enrichments of DEGs 

**Figure 4:**
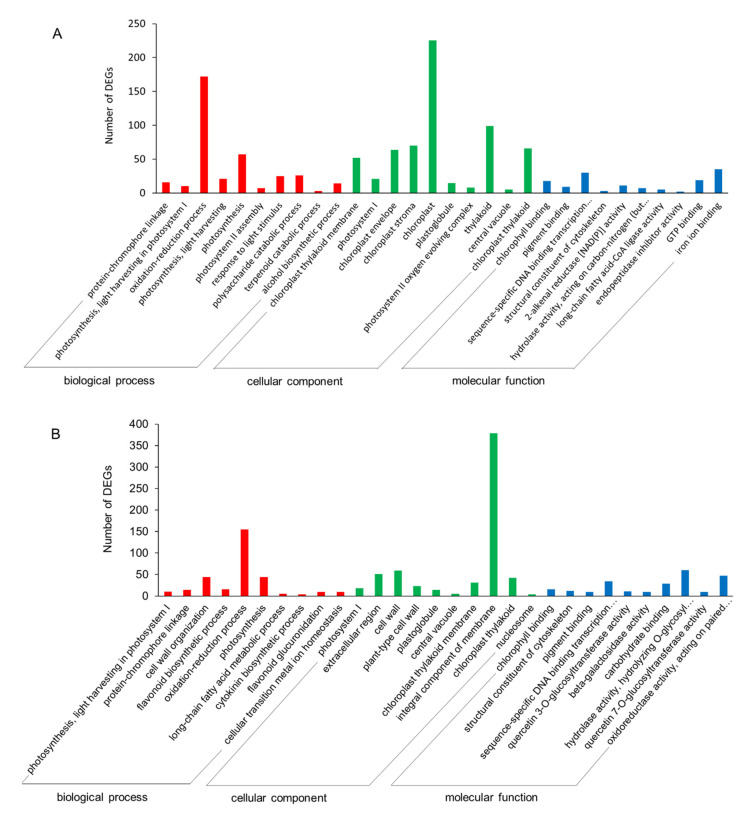

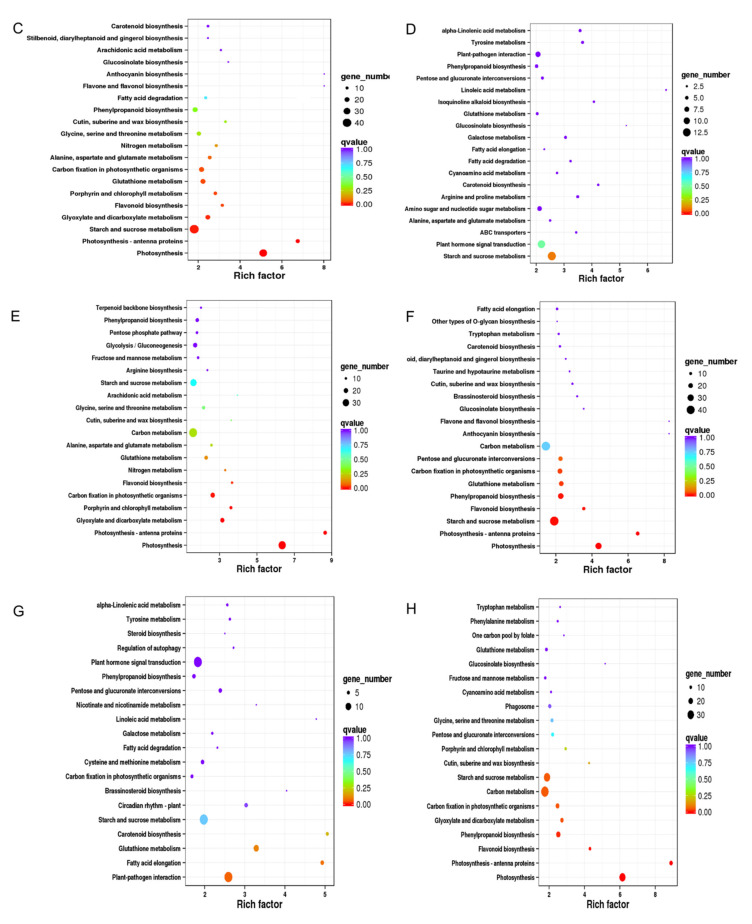
GO and KEGG enrichments of the DEGs. (**A**) GO enrichment of the DEGs in leaf blade; (**B**) GO enrichment of the DEGs in petiole; (**C**) KEGG enrichment of all DEGs in leaf blade; (**D**) KEGG enrichment of up-regulated DEGs in leaf blade; (**E**) KEGG enrichment of down-regulated DEGs in leaf blade; (**F**) KEGG enrichment of all DEGs in petiole; (**G**) KEGG enrichment of up-regulated DEGs in petiole; (**H**) KEGG enrichment of down-regulated DEGs in petiole.

GO enrichment analysis of the DEGs was performed. In leaf blade, DEGs were classified into three GO categories, namely biological process (BP), cellular component (CC), and molecular function (MF). In BP category, the top 3 items were protein-chromophore linkage (16 unigenes), photobiosynthesis and light harvesting in photosystem I (10 unigenes), and oxidation-reduction process (172 unigenes); in CC category, the top 3 items were chloroplast thylakoid membrane (52 unigenes), photosystem I (21 unigenes), and chloroplast envelope (64 unigenes); and in MF category, the top 3 items were chlorophyll-binding (18 unigenes), pigment binding (9 unigenes), and sequence-specific DNA binding transcription factor activity (30 unigenes) (Figure 4A, Table S4). In petiole, DEGs enriched in BP category were similar with those in leaf blade, exactly in cell wall organization (44 unigenes), flavonoid biosynthetic process (16 unigenes), etc; in CC category, the top 3 items were enriched in photosystem I (18 unigenes), extracellular region (51 unigenes), and cell wall (59 unigenes); in MF category, the top 3 items were also enriched in chlorophyll-binding (16 unigenes), pigment binding (9 unigenes), and structural constituent of the cytoskeleton (12 unigenes) (Figure 4B, Table S5). 

KEGG enrichment analysis showed that the DEGs in leaf blade were mainly enriched in photobiosynthesis (ko00195), photobiosynthesis-antenna proteins (ko00196), starch and sucrose metabolism (ko00500), glyoxylate and dicarboxylate metabolism (ko00630), flavonoid biosynthesis (ko00941), and porphyrin and chlorophyll metabolism (ko00860) (Table S6). The main enrichment pathways of DEGs in petiole were similar with those in leaf blade. Furthermore, DEGs in petiole were also enriched in phenylpropanoid biosynthesis (ko00940), glutathione metabolism (ko00480), and pentose and glucuronate interconversions (ko00040) (Table S7). In leaf blade, starch and sucrose metabolism, as well as plant hormone signal transduction pathway were up-regulated; photobiosynthesis, and porphyrin and chlorophyll metabolism were down-regulated. In petiole, plant-pathogen interaction, and fatty acid elongation pathway were up-regulated, while photobiosynthesis, flavonoid biosynthesis, phenylpropanoid biosynthesis, carbon metabolism, starch and sucrose metabolism were down-regulated (Figure 4C–H). These results indicated that the main enrichment pathways of DEGs in petiole and leaf blade were similar, more exactly, indicating that drought stress mainly affected the photobiosynthesis, starch and sucrose metabolism, flavonoid biosynthesis, phenylpropane biosynthesis, glutathione metabolism, and pentose and glucosaldehyde interconversion pathway in *A. sieboldii*.

#### 2.3.5. Cluster analysis of DEGs

**Figure 5:**
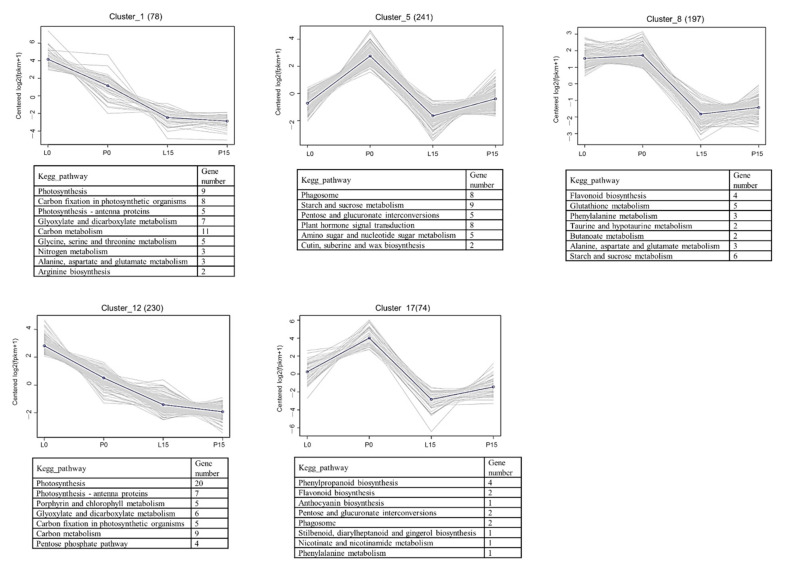
Cluster analysis of DEGs. The numbers in parentheses were total number of unigenes in the clusters. The enriched KEGG pathways with a corrected *p*-value < 1 were listed in each cluster.

All of the 4453 DEGs were clustered to identify the common expression patterns, as finally led to 17 profiles (Figure 5 and Figure S1). Nine clusters were down-regulated in drought both in leaf blade and petiole, while the cluster 2, 7, 9, 10, 14 and 16 were up-regulated. In order to identify the pathways in each cluster, the KEGG enrichment was analyzed, and only the pathways with a corrected *p*-value < 1 were listed. In cluster 1 and cluster 12, DEGs were enriched in photobiosynthesis, containing nine and twenty unigenes, respectively; the clusters showed a clearly down-regulated trend both in leaf blade and petiole. In cluster 12, five unigenes were annotated to porphyrin and chlorophyll metabolism pathway, of which the down-regulation was well related to the decrease in chlorophyll content. In cluster 8 and cluster 17, flavonoid biobiosynthesis and phenylpropanoid biobiosynthesis pathways were mostly enriched, and both pathways showed down-regulated expression. In cluster 9, six unigenes annotated to starch and sucrose metabolism pathway were up-regulated. However, an opposite trend was seen in cluster 11, with ten down-regulated unigenes. In cluster 14, two unigenes annotated to isoquinoline alkaloid biosynthesis pathway, and two unigenes annotated to tyrosine metabolism were up-regulated. Interestingly, in *A. sieboldii*, the biosynthesis of aristolochic acid I, a toxic constituent, was predicted in these two pathways. The up-regulation of these genes would help to understand the genes involved in aristolochic acid I biosynthesis in *A. sieboldii* under the drought stress.

#### 2.3.6. DEGs Related to Photobiosynthesis, Chlorophyll and Hormone Signal Transduction

**Figure 6:**
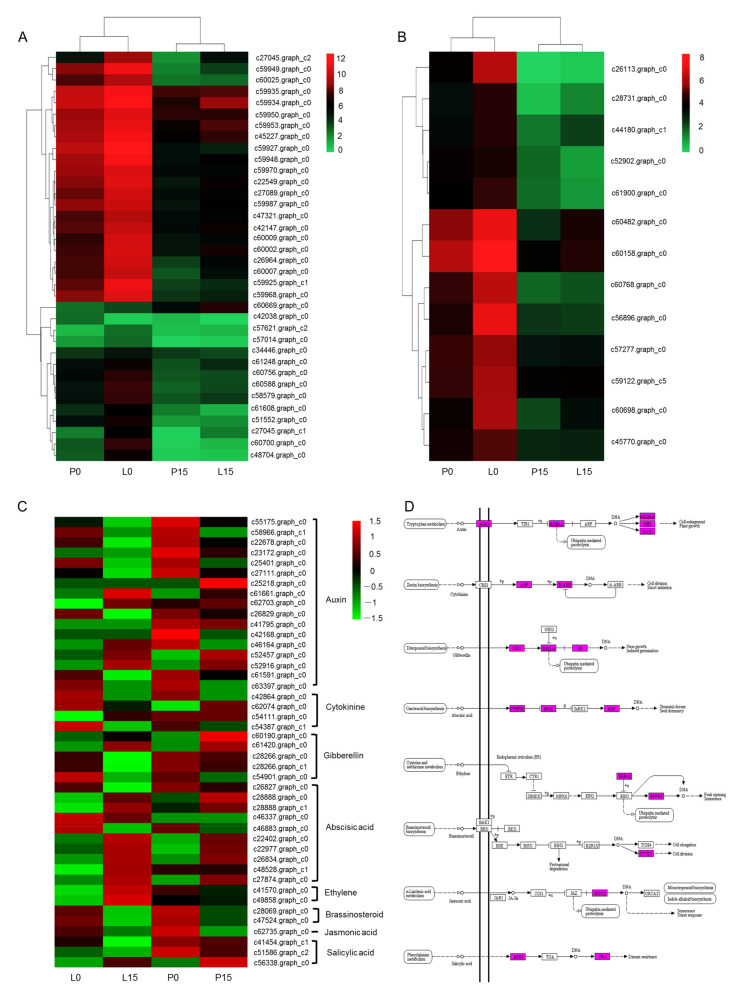
Thermal cluster analyses of DEGs in photobiosynthesis (**A**), porphyrin and chlorophyll metabolism pathway (**B**), and plant hormone signal transduction pathway (**C**,**D**). The blue color in (**D**) indicated the annotated enzymes.

In this study, 36 DEGs were annotated to the photobiosynthesis pathway (ko00195) (Figure 6A, Table S14). In leaf blade, all the unigenes were down-regulated, except for the unigene c60669.graph_c0 that was up-regulated; and in petiole, most of the unigenes were down-regulated, along with up-regulation of several unigenes including the c60669.graph_c0. In other words, the c60669.graph_c0 appeared to be kept up-regulation in both leaf blade and petiole. Indeed, the c60669.graph_c0 was functionally annotated as F-type H^+^-transporting ATPase subunit delta (ATP synthase). Among the 36 DEGs, c59927.graph_c0 showed the highest fold alteration (log_2_FC value of 8.45), with the decrease in FPKM values from 8169.93 to 25.88. Another unigene, c59927.graph_c0, annotated as an encoding gene of plastocyanin (a copper-binding protein responsible for electron transfer in photosystem I), was also down-regulated in drought condition. 

13 DEGs were annotated to porphyrin and chlorophyll metabolism pathway (ko00860) (Figure 6B, Table S15). All these genes were down-regulated in leaf blade, and the c26113.graph_c0 showed the most dramatic folding changes (FPKM values varying from 129 to 0.75 after drought treatment). Function annotation indicated that the c26113.graph_c0 encoded the chlorophyllase. The variation in expression level of c26113.graph_c0 was in accordance with that of chlorophyll content, which was reduced from 3.169 ± 0.067 mg/g F.W to 2.178 ± 0.145 mg/g F.W. 

44 DEGs were annotated to the plant hormone signal transduction pathway (ko04075) (Table S16). Most of these genes were involved in auxin and abscisic acid signal transduction. In auxin signaling pathway, four unigenes (c25401.graph_c0, c22678.graph_c0, c27111.graph_c0, c23172.graph_c0) annotated as auxin-responsive protein IAA were down-regulated; otherwise, three unigenes (c25218.graph_c0, c61661.graph_c0, c62703.graph_c0) annotated as auxin-responsive GH3 were up-regulated. Both c52457.graph_c0 and c52916.graph_c0, two of the eight unigenes that were annotated to SAUR family proteins, were up-regulated. In abscisic acid signaling pathway, five unigenes were annotated to abscisic acid receptor PYR/PYL, two of which (c28888.graph_c0 and c28888.graph_c1) were up-regulated. Four unigenes annotated as the encoding genes of protein phosphatase 2C (PP2C) were also up-regulated. The unigene c27874.graph_c0, which was annotated to the ABA-responsive element binding factor (ABF), was up-regulated in petiole (Figure 6C,D).

#### 2.3.7. DEGs Associated with Methyleugenol Biobiosynthesis 

**Figure 7:**
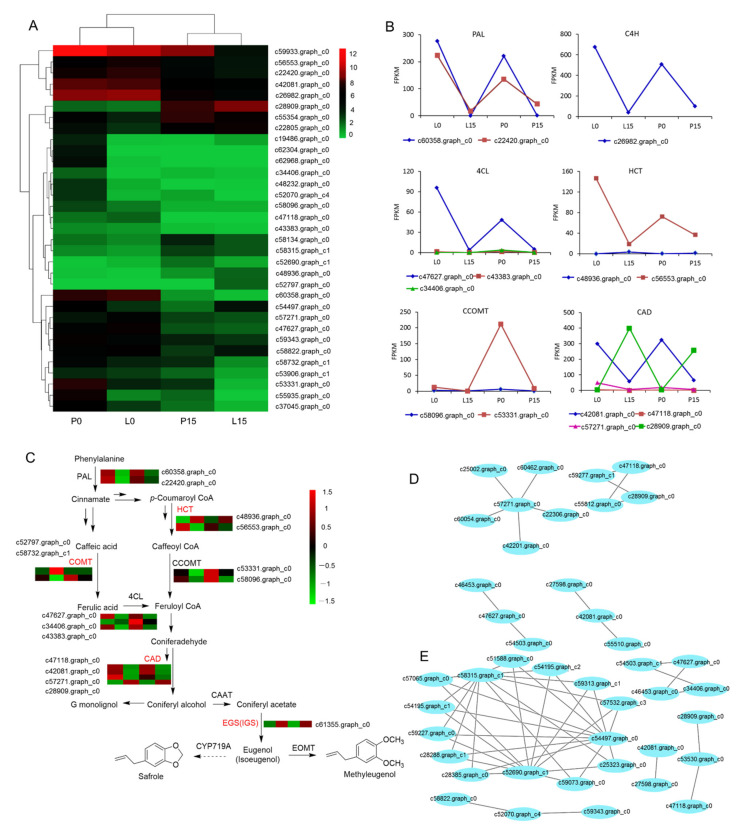
DEGs involved in methyleugenol biobiosynthesis pathway. (**A**) DEGs in the phenylpropanoid metabolic pathway. (**B**) FPKM values of DEGs related to methyleugenol biobiosynthesis. (**C**) The proposed pathway for methyleugenol biobiosynthesis in *A. sieboldii*. The four colored boxes from left to right represent the FPKM values of the unigenes in L0, L15, P0 and P15, and unigenes with red color represent up-regulated. The enzymes are L-phenylalanine ammonia-lyase (PAL), shikimate hydroxycinnamoyl transferase (HCT), caffeic acid *O*-methyltransferase (COMT), caffeoyl-CoA *O*-methyltransferase (CCOMT), *p*-coumarate CoA ligase (4CL), cinnamyl alcohol dehydrogenase (CAD), coniferyl alcohol acetyl transferase (CAAT), eugenol synthase (EGS), ioseugenol synthase (IGS), eugenol *O*-methyltransferase (EOMT), and cytochrome P450 subfamily members (CYP719A). (**D**,**E**) Interaction network of differentially-expressed proteins in phenylpropanoid metabolic pathway in leaf blade and petiole. The interaction of proteins was conducted in open-source STRING database v10.5 (https://string-db.org/, accessed on 24 July 2021) [37], and visualized with Cytoscape software v3.6.1 [38]. The annotated unigenes are listed in Supplementary Materials Table S13.

Among the total 33 DEGs that were annotated in the phenylpropanoid metabolic pathway (ko00940) in leaf blade and petiole, 14 DEGs were related to the methyleugenol biobiosynthesis. Whereas, other 19 DEGs were encoding for caffeic acid 3-O-methyltransferase [EC:2.1.1.68] (2 genes), β-glucosidase [EC:3.2.1.21] (8 genes), and peroxidase [EC:1.11.1.7] (9 genes) accordingly. Thermal cluster analysis of these 33 DEGs showed that these four tissue samples (L0, P0, L15, P15) were clustered into two branches, namely the control group (L0, P0) and drought-treated group (L15, P15). It was clearly showed that most DEGs were down-regulated (Figure 7A, Table S10). The FPKM value of 14 genes involved in methyleugenol biobiosynthesis was analyzed, and 13 of these genes were down-regulated; however, the gene c28909.graph_c0, encoding cinnamyl-alcohol dehydrogenase (CAD), showed up-regulated trend in leaf blade and petiole (Figure 7B).

In leaf blade, five DEGs were up-regulated. These unigenes were annotated to encode β-glucosidase (c22805.graph_c0), caffeic acid 3-O-methyltransferase (c52797.graph_c0), cinnamyl-alcohol dehydrogenase (c28909.graph_c0), peroxidase (c55354.graph_c0), and shikimate o-hydroxycinnamoyltransferase (c48936.graph_c0), respectively. Among them, c28909.graph_c0, c55354.graph_c0 and c48936.graph_c0 were also up-regulated in petiole. These genes are at the upstream of monolignol biobiosynthesis pathway, which is shared by the biobiosynthesis of phenylpropenes including methyleugenol [22]. In these up-regulated genes, c28909.graph_c0 (CAD) showed a highest fold change, implying that it might heavily influence the production and accumulation of methyleugenol.

Eugenol synthase (EGS), an enzyme responsible for the biosynthesis of eugenol, was searched out in the transcriptome sequencing library. It was found that a unigene (c61355.graph_c0) annotated as *isoeugenol synthase* 1 (*IGS*1), was up-regulated in both leaf blade and petiole under drought stress. Additionally, it took a key position at the last two steps in methyleugenol biosynthesis pathway (Figure 7C). We deduced that the up-regulation of *EGS*/*IGS* would promote the biosynthesis of eugenol and methyleugenol. All of the DEGs annotated to the methyleugenol biosynthesis pathway were shown in Figure 7C. 

Additionally, in leaf blade, four unigenes annotated as CAD-encoding genes, and one unigene as 4CL-encoding gene were detected. Both the CADs and 4CL could interact with other proteins. For example, CAD may interact with alcohol dehydrogenase class-P ([EC:1.1.1.1]), 2-oxoisovalerate dehydrogenase E1 component alpha subunit ([EC:1.2.4.4]), Non-functional NADPH-dependent codeinone reductase 2, and Enoyl-(Acyl carrier protein) Reductase (secoisolariciresinol dehydrogenase). Likewise, 4CL may interact with long-chain acyl-CoA synthetase [EC:6.2.1.3] (Figure 7D, Table S11). 

Moreover, in petiole, 10 unigenes were annotated as β-glucosidase (5 unigenes), CAD (3 unigenes) and 4CL (2 unigenes). β-glucosidase interacted with rac-like GTP-binding protein RAC2, tyrosine-sulfated glycopeptide receptor 1 (leucine-rich receptor-like protein kinase family protein isoform 2), LRR receptor-like serine/threonine-protein kinase GSO1, mitogen-activated protein kinase 15, and septum-promoting GTP-binding protein 1. The protein 11-β-hydroxysteroid dehydrogenase 1B (Enoyl-(Acyl carrier protein) reductase) and alcohol dehydrogenase [EC:1.1.1.1] may interact with CAD. Protein Long-chain acyl-CoA synthetase [EC:6.2.1.3] may interact with 4CL (Figure 7E, Table S12).

#### 2.3.8. DEGs Encoding Transcription Factors 

**Figure 8:**
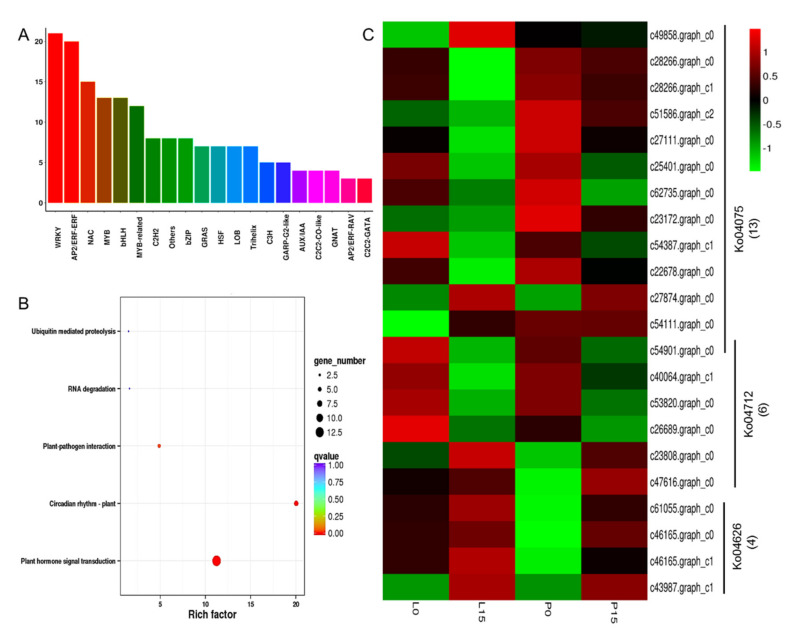
Transcription factors involved in drought stress response. (**A**) Total number of differentially-expressed transcription factors. (**B**) KEGG enrichment of the differentially-expressed TFs. (**C**) Thermal cluster analysis of the TFs in plant hormone signal transduction, circadian rhythm-plant, and plant-pathogen interaction pathway.

A total of 213 differentially expressed unigenes, annotated as transcription factors (TFs), were identified. The largest family was the WRKY, which comprised 21 unigenes and made up to 9.86% of all annotated TFs. Two of these WRKY TFs were down-regulated. 20 unigenes were annotated to AP2/ERF family, including three down-regulated unigenes. There were 15 unigenes annotated to NAC family, three of which were down-regulated. 25 unigenes were annotated as MYB and MYB-related TFs. 13 unigenes were annotated to bHLH TFs. Other transcription factors such as C2H2 (8), bZIP (8), GRAS (7), and HSF (7) were also annotated (Figure 8A, Table S8). 

KEGG enrichment of these differentially-expressed TFs indicated that they were mostly enriched in plant hormone signal transduction pathway (ko04075, 13 unigenes), plant’s circadian rhythm (ko04712, 6 unigenes), and plant-pathogen interactions (ko04626, 4 unigenes) (Figure 8B). Thermal cluster analysis of the DEGs (22 unigenes) in these three pathways showed that only three unigenes were up-regulated in leaf blade, i.e., c43987.graph_c1, c49858.graph_c0, and c54111.graph_c0, which belonged to WRKY transcription factor, AP2 domain containing ethylene-responsive transcription factor and Myb-like transcription factor, respectively. In petiole, seven unigenes were up-regulated, including four WRKY transcription factors (c61055.graph_c0, c46165.graph_c0, c46165.graph_c1, c43987.graph_c1), one bZIP transcription factor (c27874.graph_c0), one Myb-like (c47616.graph_c0), and one response regulator receiver domain containing regulator (c23808.graph_c0) (Figure 8C, Table S9).

#### 2.3.9. SSR Marker Analysis 

Totally, 5466 SSRs (Simple sequence repeats) were searched out in the present transcriptome dataset (Table S17), which can be distinguished into six types including single nucleotide, dinucleotide, trinucleotide, tetranucleotide, pentanucleotide, and hexanucleotide motifs. The MISA software was employed to analyze the SSRs in length of over 1 kb [39]. 

**Figure 9:**
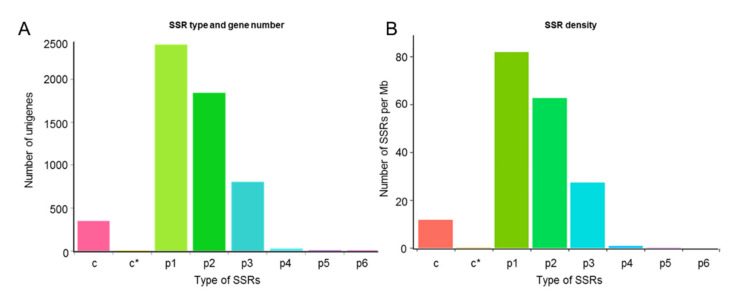
Predicated SSR markers of *A. sieboldii*. (**A**) Types and numbers of SSR markers in petiole and leaf blade of *A. sieboldii*. (**B**) SSR density in petiole and leaf blade of *A. sieboldii*. p1 to p6, six types of SSR markers; c, compound repeat SSRs; c*, compound repeat SSRs with overlap.

2410 unigenes were discovered containing single nucleotide repeats, which belonged to the most common type of the repeated motifs, accounting for 44.09% of all motifs, followed by two- (1846, 33.77%), three- (807, 14.76%), four- (30, 0.55%), six- (10, 0.18%), and five-nucleotide (6, 0.11%) repeat motifs. Besides, there were 357 complex SSRs, accounting for 6.53% of the repeat motifs. Among the dinucleotide SSRs, unigenes 509, 333, 456 and 326 contained AG, CT, GA, and TC, respectively. Therefore, AG(GA)/CT(TC) were the most frequent dinucleotide SSR motifs in *A. sieboldii*, accounting for 29.71% of all SSRs. Among the trinucleotide SSR markers, 60 unigenes contained AAG, 73 unigenes contained GAA, 72 unigenes contained TCT, 67 unigenes contained TTC, and 50 unigenes contained CTT. Therefore, AAG (GAA)/TTC(CTT) and TCT were the most frequent trinucleotide SSRs in *A. sieboldii*, accounting for 5.89% of all SSRs (Figure 9). 

### 2.4. qRT-PCR Validation 

**Figure 10:**
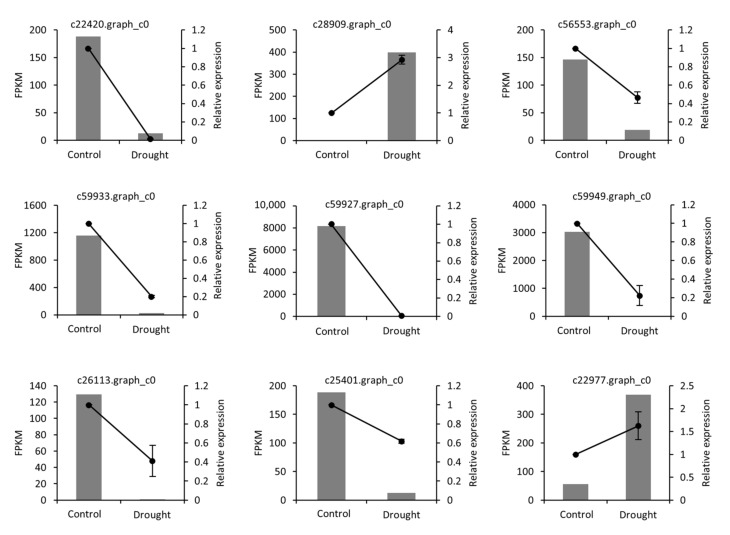
Validation of DEGs with real-time qPCR. The *Y*-axis on left side of each chart indicates FPKM value from RNA-seq. The *Y*-axis on right side indicates the relative expression level based on qRT-PCR.

To validate the transcriptome sequencing results, qRT-PCR was performed to assess the expression levels of 9 DEGs under control and drought stress. The expression trends, from RNA-Seq and qRT-PCR, showed positive correlation, thereby confirming the accuracy of DEGs data from RNA-seq (Figure 10).

## 3. Discussion

### 3.1. Morphological and Physiological Mechanisms in Response to Drought Stress in A. sieboldii

In response to drought stress, plants have developed many mechanisms either to reduce water loss or to promote water utilization efficiency, for example, changing phenotypes and functions at morphological, physiological and molecular levels [40]. Under water-deficit conditions, plants usually develop strong root system, thicker leaf blades, and smaller but denser stoma [17]. In our study, leaf blades of *A. sieboldii* exhibited a common phenotype, with drooped and rolled structure under drought. As a common and passive response of plants to water deficiency, leaf rolling is induced by the adjustment of the turgor pressure with a purpose of delaying leaf death, which reduces leaf surface, area, temperature, water consumption, and water evaporation [41]. 

It has been well known that photobiosynthesis had a large impact on plant growth. Thylakoids are the structural and foundational unit of chloroplasts for light absorption, transmission and transformation. On the other hand, photobiosynthesis is commonly affected by various biotic and abiotic environmental factors including drought stress, which can destroy the thylakoid membrane structure associated with photobiosynthesis, affect the chlorophyll content, and cause disruption of physiological processes [24]. In our study, the chlorophyll content of *A. sieboldii* was decreased under drought treatment (from 3.169 ± 0.067 mg/g FW to 2.178 ± 0.145 mg/g FW, Figure 1B), as was also seen in *Barley* whose chlorophyll content significantly declined along with the decreasing of soil water content [42]. The results of morphological and chlorophyll contents indicated that drought stress reduced chlorophyll biosynthesis and hindered photobiosynthesis in the leaf blade of *A. sieboldii*. 

Furthermore, the expression level of DEGs, annotated to porphyrin and chlorophyll metabolism pathway, was in accordance with the content of chlorophyll (Table S15). It was shown that all of the annotated DEGs were down-regulated in leaf blade. In detail, c28731.graph_c0, annotated to divinyl chlorophyllide a 8-vinyl-reductase [EC:1.3.1.75], which catalyzed the transformation from divinyl chlorophyllide to chlorophyllide, was significantly down-regulated in both leaf blade and petiole, with log_2_FC values of −3.89 and −3.47, respectively. For the chlorophyllase-encoding unigene, c26113.graph_c0 [EC:3.1.1.14], the FPKM value was hugely reduced from 129 to 0.75 in drought treatment as well as the significant changes in fold (log_2_FC value of −5.23). It was reported that the activity of chlorophyllase was related to photosystem II [43]. Besides, c28731.graph_c0 annotated as divinyl chlorophyllide a 8-vinyl-reductase, playing a key role in chlorophyllide, was down-regulated. The down-regulated expression of these genes can directly influence the chlorophyll content. It was also seen in *Atractylodes lancea* that the drought stress inhibited the photobiosynthesis, reducing the chlorophyll content [44].

Drought also limits the diffusion of CO_2_ into the chloroplasts, thereby limiting the process of photosynthetic metabolism. In our study, GO annotation analysis showed that DEGs were also enriched in photobiosynthesis, light collection in photosystem I, and redox processes. Under drought stress, a total of 36 DEGs related to photosynthetic pathway such as photosystem I subunit II, photosystem I subunit XI, F-type H^+^-transporting ATPase subunit b, photosystem I P700 chlorophyll a apoprotein A2 (chloroplast), photosystem II CP47 chlorophyll apoprotein (chloroplast), oxygen-evolving enhancer protein 1, and so on, were found in the leaf blade and petiole of *A. sieboldii*. Most of these unigenes were down-regulated except only one unigene, c60669.graph_c0, which was up-regulated and encoded F-type H^+^-transporting ATPase subunit delta, involving in oxidative phosphorylation (ko00190) and photobiosynthesis (ko00195). Our results were agreed with the gene expression model of photobiosynthesis in plants, responding to drought stress [45,46]. The profiles of DEGs involved in photobiosynthesis suggested that photobiosynthesis’s functions were significantly affected by drought at the transcriptional level. Overall, our results showed that both photobiosynthesis and chlorophyll metabolism were inhibited, which can be clearly seen from the chlorophyll content, morphology, and the expression of DEGs in photobiosynthesis and the pathway of chlorophyll metabolism.

It is known that photobiosynthesis comprises many redox reactions, in which NADP^+^ acts as an electron carrier. The drought reduces NADP^+^ by the inhibition of Calvin-Benson cycle, leading to the generation of ROS (reactive oxygen species) to transfer excess electrons to oxygen. Therefore, photosynthetic electron transport system is the major source of ROS. Oxidative stress commonly occurs in drought condition. Aerobic metabolism is often accompanied by generation of ROS, and by-products such as ^1^O_2_, H_2_O_2_, O_2_^−^, and OH• [47,48]. Under normal circumstances, ROS remain under dynamic equilibrium, whereas, under drought stress, this dynamic equilibrium gets disturbed, causing overabundance of ROS in a biological system. When ROS content exceeded the capacity for scavenge, lipid peroxidation in biological membranes increases, and consequently leads to disturbance of the physiological processes in a cell [49]. To protect cells to avoid from the injury of excessive ROS, plants have triggered a complex enzymatic and non-enzymatic antioxidant defense mechanisms to maintain the balance of the intracellular redox state. MDA is one of the final products of oxidative modification of lipids, which is increased due to lipid peroxidation and drought stress [50]. Our study showed that the MDA contents increased in both leaf blade and petiole under drought treatment, indicating that self-defense of *A. sieboldii* has been stimulated. 

Plants have evolved an antioxidant defensive system to scavenge ROS and alleviate cellular damage. This system includes protective enzymes such as superoxide dismutase (SOD), peroxidase (POD), catalase (CAT), and ascorbate peroxidase (APX) [51,52]. It is known that increase in activity of enzymes such as POD, CAT and SOD can enhance stress tolerance in plants [53,54]. Similarly, in the present study, the enzyme activities of POD and SOD were analyzed under the drought treatment. We found 12 unigenes annotated to SOD, and 65 unigenes annotated to POD (Table S18). Among the SODs, most of them showed normal expression. However, most of PODs were down-regulated, but 3 unigenes (c55354.graph_c0, c58134.graph_c0 and c54718.graph_c0) encoding POD were up-regulated. The down-regulation of theses DEGs was found to be time dependent, showing enhanced expression at first and then decreased. Further study is needed to investigate the expression of the genes encoding SOD and POD by shortening the drought stress time. Taken together, we observed that in response to drought stress, *A. sieboldii* had gone through extensive POD and SOD activity, and eliminated the excessive accumulation of ROS by up-regulated POD encoding gene expression to protect it from drought damage.

### 3.2. Effect of Drought Stress on Methyleugenol Biobiosynthesis in Leaf of A. sieboldii

Drought is one of the important environmental factors that affect the biosynthesis of natural products. For example, the essential oil biosynthesis of medicinal plants can be significantly impacted by environmental factors including drought [55,56]. The effect of drought stress on essential oil amount and composition is speculated to attribute to the perturbance on several key enzyme activities in the metabolism pathway [57]. It was reported that drought increased the contents of methylchavicol, methyleugenol, β-Myrcene, and α-bergamotene in essential oil of basil. Correspondingly, it also raised the expression levels of *CVOMT* and *EOMT*, which were the final step enzymes accordingly in the methylchavicol and methyleugenol biosynthesis pathways [20]. *A. sieboldii* produces essential oils as its main pharmacological component; however, either the effect or the mechanism of drought stress on its essential oils has remained unclear yet. In our study, under drought treatment the contents of methyleugenol and safrole were increased while the content of myristicin was decreased. A down-regulated trend was detected for the expression levels of the genes encoding trans-cinnamate 4-monooxygenase (C4H), 4-coumarate-CoA ligase (4CL), shikimate o-hydroxycinnamoyltransferase (HCT), caffeic acid 3-O-methyltransferase (COMT), cinnamyl-alcohol dehydrogenase (CAD), whereas a up-regulated trend was observed for eugenol/isoeugenol synthase (EGS/IGS)-encoding gene. EGS/IGS catalyzed the formation of eugenol/isoeugenol, which was the substrate for biosynthesis of methyleugenol/methylisoeugenol and safrole/isosafrole [58]. Therefore, the up-regulation of EGS/IGS was considered playing a vital role in increasing methyleugenol content. We also discovered the interacting protein in the biosynthetic pathway of methyleugenol. It was found that existed the interactions between CAD and other proteins, such as alcohol dehydrogenase class-P, 2-oxoisovalerate dehydrogenase E1 component alpha subunit, Non-functional NADPH-dependent codeinone reductase 2, Enoyl-(Acyl carrier protein) Reductase (secoisolariciresinol dehydrogenase), and CCR (cinnamoyl-CoA reductase) [59]. 4CL was also observed interacting with long-chain acyl-CoA synthetase. These phenomena are needed to be further verified in the future to reveal the protein interaction in response to drought. 

### 3.3. The Transcription Factors and Plant Hormones Responsive to Drought Stress

Transcription factors (TFs) widely exist in plants, which regulate gene expression and signal transduction, involving in various biological processes and biosynthetic path-ways. For example, TFs can help plants to response to abiotic stresses by activating or inhibiting gene expression at the transcription level [60]. Numbers of researches have reported that TFs such as MYB, MYC, and WRKY, played key roles in plant drought tolerance via ABA-dependent or ABA-independent pathways [34,61]. In the 213 differentially-expressed transcription factors identified in the current study, WRKY took up the most abundance, followed by AP2/ERF family. WRKY is a group of proteins that can be modulated by several mechanisms such as miRNA-mediated post-transcriptional silencing, reactive oxygen species (ROS) signaling, DNA methylation, and posttranslational modifications of histones [62]. The AP2/ERF family has also emerged as key regulators of stress responses, and it regulates lots of abiotic stresses such as heat, drought, salinity, and cold. AP2/ERF family is expressed at low levels under normal growing conditions, whereas their expressions can be sharply induced by hormones and abiotic stresses [63]. It was reported that AP2/EREBPs, WRKYs, bHLHs, and NACs were highly up-regulated in H471 genotype rice under drought stress [35]. Additionally, these TFs are key regulators of ABA-mediated stomatal closure and drought responses [64]. In soybean, overexpression of *GmWRKY12* enhanced drought tolerance and decreased malondialdehyde (MDA) content [65]. The AP2/ERF type TF GmERF3 in soybean was found to be induced by drought and abscisic acid (ABA). The overexpression of *GmERF3* in tobacco increased its tolerances to salt and drought stresses [66]. In apple, overexpression of AP2/EREBP transcription factor MdSHINE2 increased drought resistance by regulating wax biobiosynthesis [67]. In our results, we found 20 unigenes annotated to AP2/ERF family, and these TFs showed drought response. These candidate AP2/ERF TFs can be further elucidated for their roles in drought stress tolerance.

Plant hormones play important roles in plant growth and response to environmental stress. Among all the phytohormones associated with plant signaling, ABA is recognized to be most closely linked to dehydration, salt and cold [68]. ABA can decrease drought damage and enhance the drought tolerance by regulating the drought-related genes [69]. Several proteins responsible for ABA-mediated drought tolerance, including pyrabactin resistance 1 (PYR1), regulatory component of the ABA receptor (RCAR), protein phosphatase 2C (PP2C), and SnRK2s, were carefully investigated [70]. It was known that drought could trigger the production of ABA in leaf of plant, as would cause stoma closure and eventually restrict cellular growth [71,72]. PP2C are known to be the important signal mediators, which negatively regulate ABA signaling [73]. Moreover, it was reported that 269 PP2C were identified, of which 1 gene was notably down-regulated (Cluster-17196.15336(-2.2994)), and 57 SnRK2 were identified, of which 3 was significantly up-regulated under drought stress, in sea buckthorn (*Hippophae rhamnoides* ssp. *Sinensis*) [35]. Similarly, in *A. sieboldii* four unigenes were annotated to PP2C. They were differentially expressed either in petiole or leaf blade. The unigene c27874.graph_c0 annotated to SnRK2 was up-regulated, which could be inactivated by PP2C through direct dephosphorylation. Thus, it indicate that the up-regulation of SnRK2 can induce stomatal closure and maintain normal survival for *A. sieboldii*. Through activating ABA-dependent signaling pathways, the drought stress was responded in *A. sieboldii*.

Auxin is a key regulator for plant development. It influences cell division, cell elongation and programmed cell death, and drives embryonic and post-embryonic development. Moreover, its function can be influenced by drought stress [74,75]. Auxin-responsive factors (ARFs) are a group of transcription factors that can act as either activators or repressors of the transcription of auxin-inducible genes [63] so that trigger the up- or down-regulation of AUX/IAA as well as GH3 and SAUR, leading to cell enlargement and plant growth. It was reported that Aux/IAAs negatively regulated the transcription of ARFs through binding to them [76,77]. In drought stressed maize, 13 differentially expressed ARF genes were identified [78]. ARFs regulate auxin responsive genes and improve drought tolerance [79]. In our study, auxin-responsive factors, including IAA, GH3 and SAUR, were found and were differentially expressed. This suggested that ABA and other hormone signaling pathways such as Auxin were interwoven with each other to regulate the responses of *A. sieboldii* to drought to maintain normal growth or development. 

### 3.4. SSR Markers of A. sieboldii Genome

Nowadays, molecular markers play an important role in plant breeding. SSR is a highly useful tool for studies on genetic diversity, systematic and evolutionary relationship, and molecular-marker assisted breeding of plants. Due to their abundance, high polymorphism and excellent reproducibility, SSR markers are a useful source for constructing high-density genetic maps and identifying plant trait loci [80,81]. However, SSR markers are still insufficient in *A. sieboldii*. Based on the transcriptome sequencing data, a total of 5466 SSRs were preliminarily identified in this medicinal herb. Among them, AAG (GAA)/TTC(CTT) and TCT were the most abundant trinucleotide SSR marker, showing perfect accordance with the situations in oak tree [82] and castor bean [83], wherein the most abundant dinucleotide and trinucleotide motifs were AG/TC and AAG/TTC, respectively. These identified SSRs will be a valuable resource for gene mapping and analysis of the various traits in *A. sieboldii* in the future.

## 4. Materials and Methods

### 4.1. Plant Materials and Drought Treatment

Two-year-old individuals of cultivated *A. sieboldii* that were originally grown in Hanzhong, Shanxi province, were planted in the greenhouse of our laboratory. Plants were grown in plastic pots. The soil was mixed with vermiculite and perlite. The growth conditions were as follows: 16 h/8 h (light/dark) at 25 °C, with 45% relative humidity and 200 µmol m^−2^ s^−1^ light intensity. Plants at the flowering stage were used for drought treatment experiments. In control group, the individuals were irrigated once a week, while in drought treatment group, the ijndividuals remained non-irrigation for 15 days. The petioles and leaf blades were collected separately from each individual, and then weer frozen immediately in liquid nitrogen and stored at −80 °C before use.

### 4.2. Determination of Chlorophyll and MDA Contents 

The chlorophylls in leaf blade were extracted and detected spectrophotometrically as described [84]. Briefly, 0.1 g plant tissues were ground in liquid nitrogen, followed extraction with 95% ethanol for several times until the plant fiber was colorless; then, 95% ethanol was added to the solution to a volume of 10 mL. The absorbance of the solution at 649 nm and 665 nm were measured. The contents of chlorophyll a and b were calculated, using formula chlorophyll a = 13.95A_665_–6.88A_649_ and chlorophyll b = 24.96A_649_–7.32A_665_. The total chlorophyll content was the sum of chlorophyll a and chlorophyll b. MDA content in leaf blades and petioles was detected with MDA kits (Solarbio Science, Beijing, China). Absorbances at 450 nm, 532 nm, and 600 nm were detected by using a fluorescence spectrophotometer (Varioskan Flash, Germany).

### 4.3. Determination of SOD and POD Activities

SOD and POD activities were determined with a commercially available SOD kit and POD kit (Solarbio Science, Beijing, China), according to the instructions. For SOD assay, absorbance at 560 nm was detected and used for calculating inhibition rate; when the inhibition rate of xanthine oxidase coupling reaction reached 50%, it was defined as one SOD enzyme activity unit. For POD assay, absorbances at 470 nm were detected either at 30 s or at 2 min 30 s after the reaction started; when the variable of absorbance at 470 nm per minute per gram of sample reached 0.01, it was defined as one enzyme activity unit. Both SOD and POD enzyme activities were displayed as U/g fresh weight plant samples.

### 4.4. GC-MS Analysis of the Major Volatile Bbioactive Constituents

For GC-MS analysis, 0.5 g petioles were cut into small pieces and grind with liquid nitrogen. Samples were transferred into a 40 mL headspace bottle. We used the SPME method for the extraction of the volatile component. The samples were balanced in a 60 °C water bath for 10 min, then the SPME needle tube was inserted into the headspace bottle. After the extraction head was fixed, the headspace bottle was stirred with a speed of 1000 rpm/min. After extraction for 30 min, the volatile component was quickly inserted into the injection port of the gas chromatograph and absorbed for 5 min. The volatile component adsorbed on the coating film of the SPME fiber head could be rapidly pyrolyzed and identified by GC-MS. 

GC-MS analysis was performed, using Agilent 7890B-7000D instrument with an Agilent 122-5532UI column (30 m × 250 μm × 0.25 μm). The temperature conditions were as follows: 50 °C for 1 min; heating from 50 °C to 100 °C at a rate of 10 °C/min; heating from 100 °C to 160 °C at a rate of 2 °C/min; heating from 160 °C to 300 °C at a rate of 10 °C/min; then, holding at 300 °C for 5 min. The carrier gas, helium, was adjusted to 1 mL/min, with the split ratio and split flow of 5:1 and 5 mL/min ac ordingly. Ion source temperature was 250 °C, and the ionization energy was 70 eV with a mass-scan range of 33–500 AMU. Compounds were identified by matching their mass data with the NIST mass spectra database and analyzed with the help of the MSD Chemstation software.

### 4.5. RNA Extraction, Transcriptome Sequencing, and de Novo Sequence Assembly

Total RNAs from the collected petiole and leaf blade were extracted, using TRIzol^®^ reagent (Invitrogen, Waltham, MA, USA) in accordance with the manufacturer’s instructions. The integrity of the extracted RNA was assessed via agarose gel electrophoresis. The quantity was determined with a NanoDrop ND-1000 spectrophotometer (Thermo Scientific, Waltham, MA, USA). The quality of the extracted RNA was assessed on Agilent Bioanalyzer 2100 system, which was acceptable for library construction with RIN values > 7.7 and the 28S:18S ratios ≥1.5.

For library preparation, 1 μg total RNA was used as input material. Sequencing libraries were generated by using NEBNext^®^Ultra™ RNA Library Prep Kit for Illumina^®^ (NEB, San Diego, CA, USA), following the manufacturer’s recommendations. The library fragments were purified with Beckman Agencourt AMPure XP (Beckman Coulter, Brea, CA, USA), and the library quality was assessed on the Agilent Bioanalyzer 2100 system. The libraries were constructed by Biomarker Biotechnology, Co., Ltd. (Beijing, China) and sequenced with the approach of Illumina Hiseq 2000 platform. Two-terminal sequencing method was used to generate paired-end reads. The paired-end reads were further assembled through Trinity software (v2.5.1) with parameters as default. The assembly results were sequenced and de-redundantly processed by using sequence clustering software (TGICL) to obtain non-redundant unigene sequences.

### 4.6. Function Annotation and DEG Analysis 

To determine the putative function of unigenes, we searched the assembled unigenes against several databases, including the non-redundant protein sequences (NR) [85], Clusters of Orthologous Groups of proteins (COG) [86], euKaryotic Orthologous Groups (KOG) [87], Swiss-Prot [88], Kyoto Encyclopedia of Genes and Genomes (KEGG) [89], Gene Ontology (GO) [90], eggNOG [91], and Protein Family (Pfam) [92] with a BLAST E-value parameter at less than 1 × 10^−5^ and the HMMER parameter E-value at less than 1 × 10^−10^. The expression values of reads were normalized with Reads Per Kilobase of exon Model per Million mapped reads. The threshold P was adjusted, using the false discovery rate (FDR) in multiple hypothesis testing. This study used the edgeR package (http://www.r-project.org/, accessed on 24 July 2021) to determine the DEGs, with the cut-off set as FDR ≤ 0.01 and |log_2_FC| ≥ 2. All the DEGs were subjected to GO function enrichment analysis via topGO R packages [93], and to KEGG pathway enrichment analysisvia KOBAS software [94]. Cluster analysis of DEGs was conducted through cluster package (https://cran.r-project.org/web/packages/cluster/index.html, accessed on 24 July 2021) [95]. Transcription factor and hormone prediction was performed by using BMKCloud (www.biocloud.net, accessed on 24 July 2021). In order to assess whether the de novo transcriptome dataset can be used to develop genetic markers for *A. sieboldii*, we searched for SSRs from the assembled sequences. SSR marker analysis was performed via MISA (http://pgrc.ipkgatersleben.de/misa/misa.html, accessed on 24 July 2021) on the platform BMKCloud (www.biocloud.net, accessed on 24 July 2021). 

### 4.7. Validation of Expression Data by qRT-PCR 

To verify the reliability of the data obtained by RNASeq, real-time qPCR experiment was carried out, in which were determined the expression patterns of nine genes responsive for phenylpropanoid biobiosynthesis, porphyrin and chlorophyll metabolism, and hormone signal transduction (Table S19). 2× ChamQ Universal SYBR qPCR master mix (Vazyme, Nanjing, China) was used in the experiment, and qRT-PCR was performed on an Applied Biosystems StepOnePlus system, with the 18S rRNA gene as the internal control. Relative expression levels were calculated by the 2^−ΔΔCt^ method [96].

### 4.8. Statistical Analysis 

Statistical analyses were performed with the SPSS software. Graphs were made by using GraphPad Prism software and Microsoft Excel. The values in each figure were the mean ± SD of three replicates. Significant differences at *p* < 0.05 (*), *p* < 0.01 (**), and *p* < 0.001 (***) were applied to all statistical tests.

## 5. Conclusions

In summary, we conducted physiological, biochemical and transcriptomic analysis of *A. sieboldii* plants under drought treatment. It was observed that drought stress decreased the chlorophyll content, but increased the activities of SOD and POD. The contents of methyleugenol and safrole in essential oil were improved under drought stress. All DEGs annotated to porphyrin and chlorophyll metabolism pathways were down-regulated, whereas three unigenes encoding POD were up-regulated. Moreover, a CAD-encoding unigene and an IGS-encoding unigene were also up-regulated, which were positively correlated with the methyleugenol content. Our study will help to understand the physiological and biological regulatory mechanisms of drought tolerance in *A. sieboldii*. Additionally, it would be a valuable resource for the metabolically engineering of these key genes, to improve the metabolic content of methyleugenol in *A. sieboldii*.

## Figures and Tables

**Table 1:** Length distribution of transcripts and unigenes.

Length Range	Transcript	Unigene
300–500	33,009 (21.91%)	18,524 (41.15%)
500–1000	36,657 (24.33%)	12,228 (27.16%)
1000–2000	43,693 (29.00%)	8635 (19.18%)
2000+	37,316 (24.77%)	5629 (12.50%)
Total Number	150,675	45,016
Total Length	218,185,217	44,921,544
N50 Length	2097	1545

**Table 2:** Function annotations of unigenes.

Database	Annotated Number	300 ≤ Length < 1000	Length ≥ 1000
COG	6226	1303	4923
GO	11,184	4030	7154
KEGG	7574	2610	4964
KOG	12,185	4455	7730
Pfam	14,251	3973	10,278
Swissprot	14,415	4988	9427
eggNOG	19,257	7278	11,979
Nr	20,366	8170	12,196
All_Annotated	20,551	8324	12,227

## Data Availability

The data presented in this study are available in Supplementary Materials, Tables S1–S17.

## Supplementary Materials

The following are available online at https://www.mdpi.com/article/10.3390/ijms222413402/s1.
