# Supplementary material for: Physiological, Biochemical and Transcriptomic Analysis of the Aerial Parts (Leaf-Blade and Petiole) of Asarum sieboldii Responding to Drought Stress
Source: Int J Mol Sci. 2021 Dec 13;22(24):13402. doi: 10.3390/ijms222413402 (PMC8708997; doi:10.3390/ijms222413402)
Supplement: Supplementary file 1 [file ijms-22-13402-s001.zip › S1 Primers and supplementary figures.pdf]

Table S19 Primers used for qRT-PCR analysis.

Primers		Sequence (5'-3')
c22420.graph_c0	F	ACTTCAGAAGGAGCTCATAAG
	R	CTTAGTGGGAGACATGGAGTG
c28909.graph_c0	F	ACACACTTGTCGATTACCTG
	R	AGCGCACGCAGAAGTAGGTT
c56553.graph_c0	F	ATGCACAACCGATCCTCTG
	R	GGATTTGAAATTCCGAGGATG
c59933.graph_c0	F	ACTCATGTCCTCAGGCAGAAG
	R	TCCTCAGCCCGAAACTCCTG
c59927.graph_c0	F	ATTACTCGCCAGCAACGCCCT
	R	ACCTCACCGGGTGCGTTCA
c59949.graph_c0	F	ATGCACCACTATCCAAGGCTGA
	R	ATTGAGCCATGGCTTCCTTCTTG
c26113.graph_c0	F	ACATGTGTACTCGACCGCA
	R	TCATTAAAGCTTACTTGGAAGG
c25401.graph_c0	F	ATGGGTGGATCTGAGGAAGT
	R	AGTCCAATGGCCTCTGATCCCTT
c22977.graph_c0	F	ATTGGCCGGAGACGGGAC
	R	AAGCTTCTCTCCATCGAC
18SrRNA	F	AACGAGACCTCAGCCTGCT
	R	CCCAGAACATCTAAGGGCA

Figure S1 The mass data of methyleugenol, safrole and myristicin.

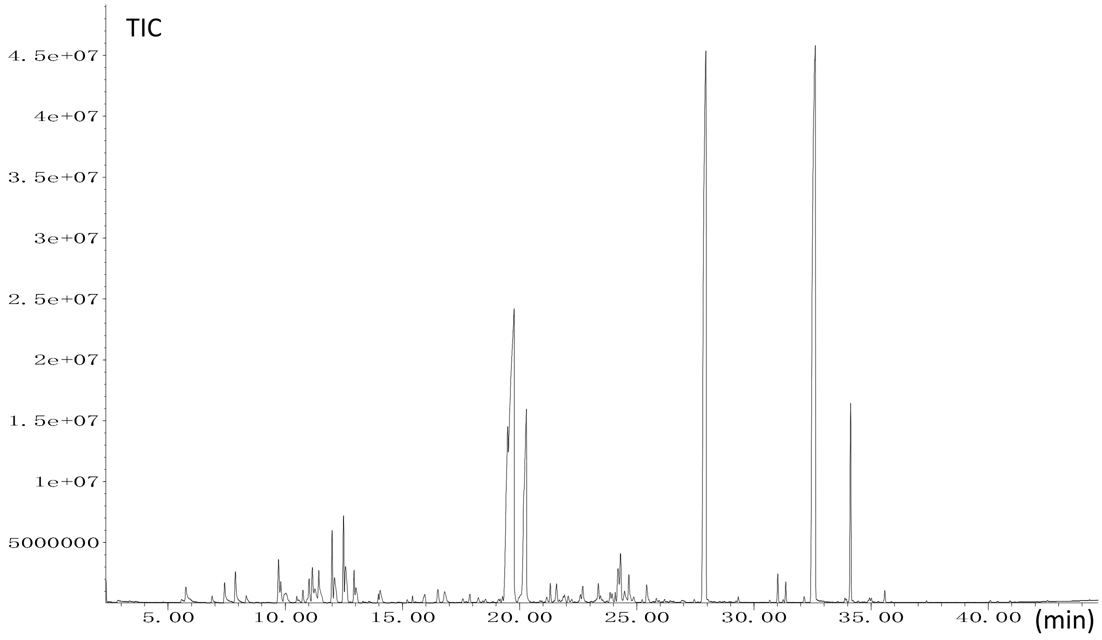

1) Methyleugenol (Rt 30.671 min)

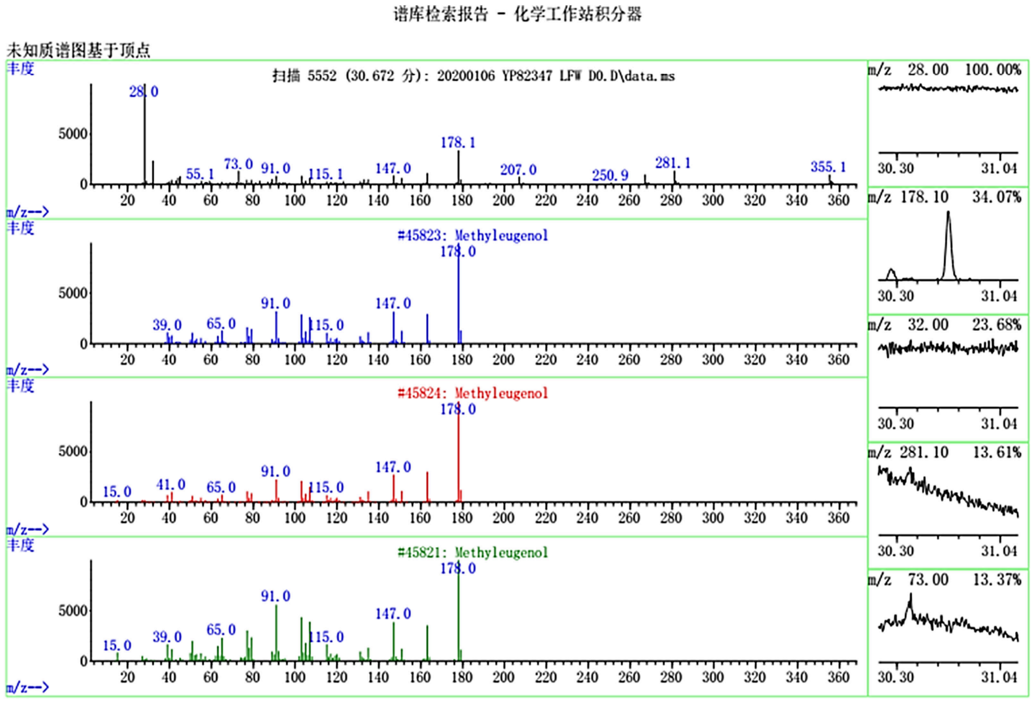

样品:

峰编号: 136 30.671 分钟处 面积: 1503368 面积 % 0.01

每个谱库中 3 个最匹配的记录。 Ref\# CAS\# 匹配度

C:\Database\NIST14.L

1 Methyleugenol	45823	000093-15-2	95
2 Methyleugenol	45824	000093-15-2	95
3 Methyleugenol	45821	000093-15-2	86

2) Safrole (Rt 27.828 min)

谱库检索报告 - 化学工作站积分器

未知质谱图基于顶点

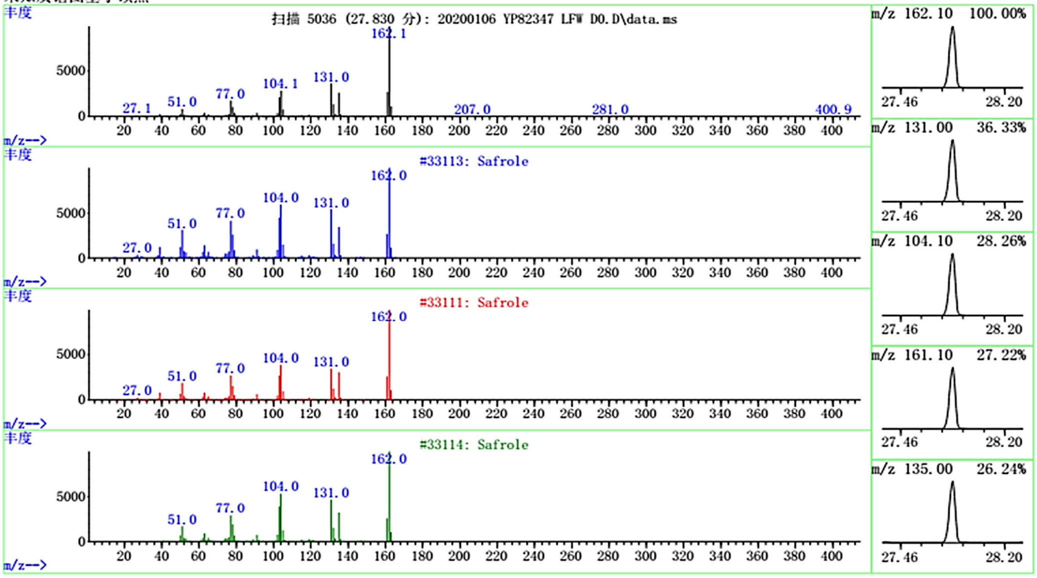

样品:

峰编号: 123 27.828 分钟处 面积: 459077264 面积 % 3.93

每个谱库中 3 个最匹配的记录。	Ref\#	CAS\#	匹配度
C:\Database\NIST14.L			
1 Safrole	33113	000094-59-7	97
2 Safrole	33111	000094-59-7	97
3 Safrole	33114	000094-59-7	96

3) Myristicin (Rt 32.574 min)

谱库检索报告 - 化学工作站积分器

未知质谱图基于顶点

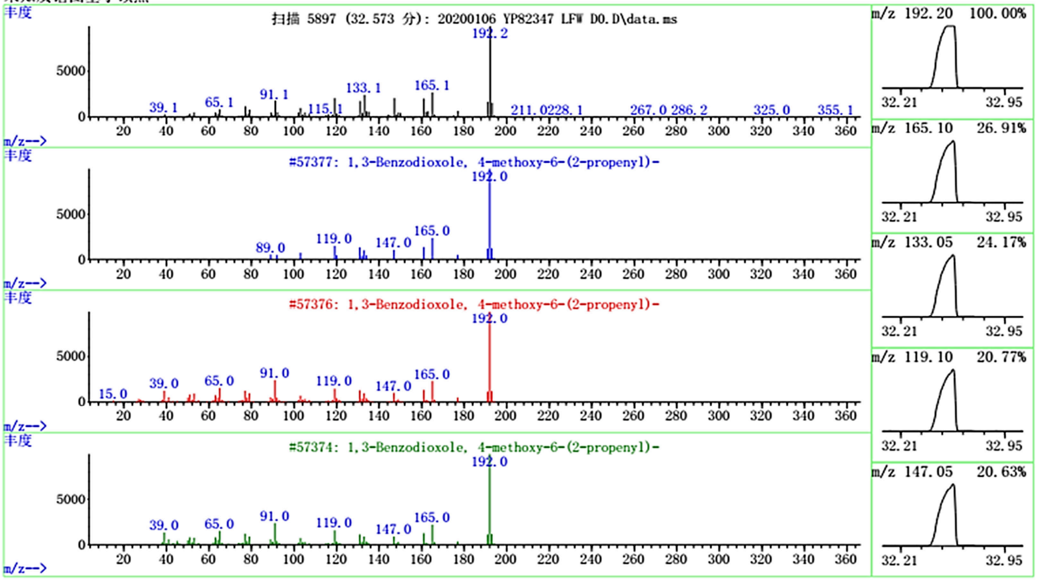

样品:

峰编号: 144 32.574 分钟处 面积: 2973819548 面积 % 25.46

每个谱库中 3 个最匹配的记录。	Ref\#	CAS\#	匹配度
C:\Database\NIST14.L			
1 1,3-Benzodioxole, 4-methoxy-6-(2...	57377	000607-91-0	96
2 1,3-Benzodioxole, 4-methoxy-6-(2...	57376	000607-91-0	96
3 1,3-Benzodioxole, 4-methoxy-6-(2...	57374	000607-91-0	95

Figure S2 Cluster analysis of DEGs.

Cluster_1 (78)

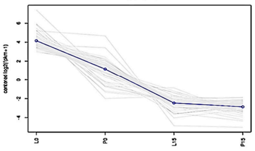

Kegg_pathway	Gene number
Photosynthesis	9
Carbon fixation in photosynthetic organisms	8
Photosynthesis - antenna proteins	5
Glyoxylate and dicarboxylate metabolism	7
Carbon metabolism	11
Glycine, serine and threonine metabolism	5
Nitrogen metabolism	3
Alanine, aspartate and glutamate metabolism	3
Arginine biosynthesis	2

Cluster_2 (160)

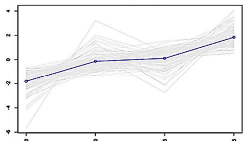

Kegg_pathway	Gene number
Glycine, serine and threonine metabolism	3
Cysteine and methionine metabolism	2
Glycolysis / Gluconeogenesis	2

Cluster_3 (446)

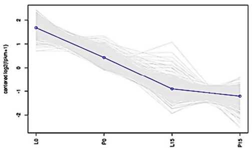

Kegg_pathway	Gene number
Glutathione metabolism	7
Porphyrin and chlorophyll metabolism	5
Ribosome	14

Cluster_4 (419)

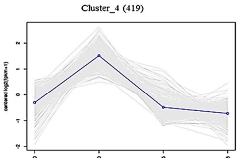

Cluster_5 (241)

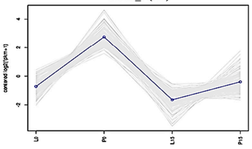

Kegg_pathway	Gene number
Phagosome	8
Starch and sucrose metabolism	9
Pentose and glucuronate interconversions	5
Plant hormone signal transduction	8
Amino sugar and nucleotide sugar metabolism	5
Cutin, suberine and wax biosynthesis	2

Cluster_6 (206)

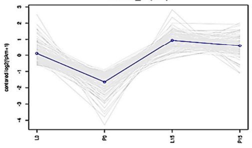

Kegg_pathway	Gene number
Plant-pathogen interaction	5
Pentose and glucuronate interconversions	3
Fatty acid elongation	2

Cluster_7 (416)

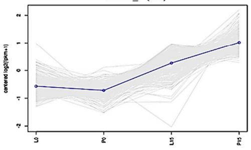

Kegg_pathway	Gene number
Circadian rhythm - plant	5
Cyanoamino acid metabolism	3
Carbon fixation in photosynthetic organisms	4
Pyruvate metabolism	4

Cluster_8 (197)

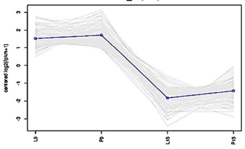

Kegg_pathway	Gene number
Flavonoid biosynthesis	4
Glutathione metabolism	5
Phenylalanine metabolism	3
Taurine and hypotaurine metabolism	2
Butanoate metabolism	2
Alanine, aspartate and glutamate metabolism	3
Starch and sucrose metabolism	6

Cluster_9 (369)

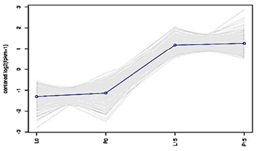

Kegg_pathway	Gene number
Starch and sucrose metabolism	6
Galactose metabolism	3

Cluster_10 (277)

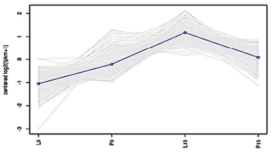

Kegg_pathway	Gene number
Linoleic acid metabolism	2

Cluster_11 (365)

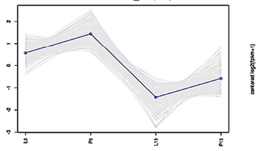

Kegg_pathway	Gene number
Starch and sucrose metabolism	10
Vitamin B6 metabolism	3
Cyanoamino acid metabolism	4
Glucosinolate biosynthesis	2

Cluster_12 (230)

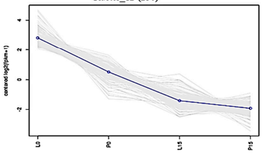

Kegg_pathway	Gene number
Photosynthesis	20
Photosynthesis - antenna proteins	7
Porphyrin and chlorophyll metabolism	5
Glyoxylate and dicarboxylate metabolism	6
Carbon fixation in photosynthetic organisms	5
Carbon metabolism	9
Pentose phosphate pathway	4

Cluster_13(219)

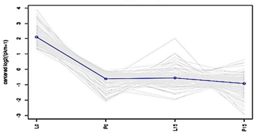

Kegg_pathway	Gene number
Vitamin B6 metabolism	2
Diterpenoid biosynthesis	2
Glyoxylate and dicarboxylate metabolism	3
Nitrogen metabolism	2

Cluster_14(118)

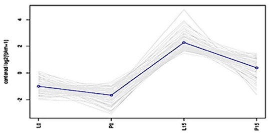

Kegg_pathway	Gene number
Isoquinoline alkaloid biosynthesis	2
Tyrosine metabolism	2
Glucosinolate biosynthesis	1
Starch and sucrose metabolism	3

Cluster_15(512)

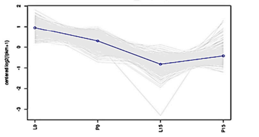

Kegg_pathway	Gene number
Cutin, suberine and wax biosynthesis	3
Fatty acid degradation	3

Cluster_16(126)

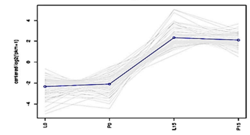

Kegg_pathway	Gene number
Plant-pathogen interaction	3

Cluster_17(74)

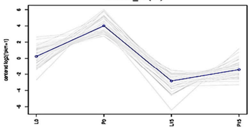

Kegg_pathway	Gene number
Phenylpropanoid biosynthesis	4
Flavonoid biosynthesis	2
Anthocyanin biosynthesis	1
Pentose and glucuronate interconversions	2
Phagosome	2
Stilbenoid, diarylheptanoid and gingerol biosynthesis	1
Nicotinate and nicotinamide metabolism	1
Phenylalanine metabolism	1
